# Geographical Differences in the Quality of Life in Poland: Challenges of Regional Policy

**DOI:** 10.1007/s11205-021-02829-x

**Published:** 2021-11-01

**Authors:** Paweł Churski, Robert Perdał

**Affiliations:** Department of Regional and Local Studies, Faculty of Human Geography and Planning, Adam Mickiewicz University, Poznań, Poland

**Keywords:** Quality of life, Economic growth, Socio-economic development, Regional policy, Poland

## Abstract

Commonly used in human geography, quality of life (QOL) refers to the way and degree to which objective human needs are met, not only existential ones, but also those regarding the development of the human personality. This article diagnoses QOL understood in this way in Poland and its changes between 2004 and 2018 (i.e. during modernization), which saw the continuation of systemic transformation after joining the EU. To fully identify the regularity of spatial differences in the analysed phenomenon, 380 local units are used as reference points. First, we formulated an operational definition of multifaceted QOL with its separate dimensions, as well as selecting and constructing organized indicators that can be determined at the local level. Then we quantified local differences in the rate of growth and the level of QOL using multivariate analysis. Third, on the basis of the obtained results, we sought to reach the most significant conclusions: (1) the dynamic economic growth, enjoyed in Poland in 2004–2018, did not change significantly the spatial diversity of the quality of life on a local level. (2) To a large extent, the persistent differences in the quality of life in Poland are related to the historical spatial differences in the level of urbanisation and the well-established, traditional economic structure of the specific territorial units. (3) A higher degree of urbanisation coupled with the service- and service and industry-related functional nature of the local units in Poland are conducive to a better quality of life. This confirms the regularities observed in the countries on the verge of a rapid economic development.

## Introduction

In social terms, an increase in quality of life (QOL) is assumed to be the primary goal of socio-economic development, including also regional growth. In the social sciences, QOL is not a definite term. Different researchers use their own definitions of this concept and emphasize its various aspects. In the geographical sciences, particular attention has been paid to spatial differences in the conditions in which people live and their personal traits related to education, skills and health as determined by these conditions; this leads to a distinction between different qualities of places to live (Burton, 2014). According to Pacione (1982), the meaning of concept of QOL in human geography is intended to refer either to the conditions of the environment in which people live (air and water pollution or poor housing for example), or to some attribute of people themselves (such as health or educational achievements). Generalizing the term, Johnston (2000, pp. 662, 763) states that QOL means “the state of social well-being of individuals or group, either as they perceive it as it is identified by ‘observable indicators’”, with social well-being understood as “the degree to which a population’s needs and wants are being met”. Today, in the social sciences, as a result of an effort to bring order to conceptual chaos, the term QOL has both a narrower and a broader meaning. In a narrower sense, it refers to the term ‘level of living’ or ‘standard of living’, which in turn is perceived as equal to social welfare. In this narrower approach, which is widely used in human geography, QOL relates to the way and degree of meeting objective human needs, not only existential needs, but also those concerning the development of the human personality (Smith, 1977). In its broader meaning, QOL contains two elements: social welfare and well-being (Cummins, 2000). The conception of well-being was developed in the area of psychology and sociology (Allardt, 1976; Campbell, 1976), but it is not strictly defined. Well-being embraces individual needs and subjective perception such as a sense of happiness, prosperity in life, satisfaction with professional and family life and awareness. Subjective well-being is mainly the study subject of pure sociology (Reig-Martínez, 2013; Russo & Terraneo, 2020).

In this article, QOL is perceived in the narrower approach used in geographical research, identifying it with the level of living determined by the possibility and assessment of satisfying both the material and immaterial needs by local inhabitants (Chojnicki & Czyż, 1987, 1992; Knox, 1973, 1994; Zborowski, 1989). This concerns, on the one hand access to jobs, housing and basic services, while on the other, it also includes needs related to the development of knowledge, qualifications and skills, health protection, development of social ties and active citizenship, as well as those related to the state of the natural environment. It should be indicated, however, that the proper diagnosis of QOL should also consider people’s subjective assessments of their possessions and the ability and state of meeting their needs. Nevertheless, an analysis of the subjective perception of QOL, which refers to the conceptions of social welfare and well-being (Aslam & Corrado, 2012), is not possible here, because of the lack of such information in the country at the local spatial level^1^ (González et al., 2011).

This article sought to identify changes in QOL at the geographical level in Poland’s local patterns. The subject of the analysis is the spatial differences in QOL in two selected conditions of Poland’s economy–that is, in the year 2004, which corresponds to the date of the country’s accession to the European Union (EU), and in 2018, which corresponds to its present socio-economic situation. In detailing the main objective, the aim is to recognize those components of QOL which are symptoms (vehicles) of significant changes in shaping the spatial differences of the socio-economic development level and, consequently, in QOL. In the first section of the article, attention is paid to the relationship between economic growth and socio-economic development, illustrated with examples of trends observed in Poland during the last three decades that embrace the transformation and modernization of the socio-economic system. Then, based on the operational definition of QOL, a model of the life quality structure is formulated, subject to further empirical concretization. The second, main part of the article, is an empirical study containing an analysis of changes in the spatial diversity of QOL in Poland by local units in the years 2004 and 2018, which was implemented according to a specific algorithm using mathematical and statistical methods (data and methods, results). In the third section (discussion), starting from the spatial distribution at the local level, an attempt has been made to determine historical and contemporary factors affecting the development of QOL.

## Background

In the process of socio-economic development, economic growth creates social changes which encompass changes in QOL (Chiappero-Martinetti et al., 2015; Ferraz et al., 2020). The nature of this influence is highly complex, varies according to the elements of QOL and relative to a particular social system in which human needs develop, subject to continuous (constant) evolution (Hult et al., 2020). This leads to a conclusion, emphasised in literature on the subject, that economic growth does not always result in developmental effects or improved QOL while its result is, to a large extent, socially diversified (Easterlin, 1974, 2001; Joshua, 2017; Kenny, 1999). What is morethe impact of economic growth is also spatially diversified in the development process and depends on specific regional and local resources making up territorial capital (Capello, 2009; Churski et al., 2021; Fernandez-Vazquez et al., 2020).

The development of the relationship between economic growth and social development can be traced in the socio-economic development process of today’s Poland from the socio-political transition in 1989 (Domański, 2005). This process began with the transformation which involved changes in the economy that resulted from the transition from a centrally planned system to a market economy. In the initial phase of the transformation (in the first half of the 1990s), in conditions of economic recession and difficulties in adaptation to a new system, what appeared were high unemployment, inflation, a systematic fall in real remuneration, the occurrence of extreme poverty and an increase in social pathologies. This had a strong negative impact on people’s QOL and resulted in growing social disparity (Bielecki, 1992). It was not only until after the first half of the 1990s, in the phase of the advanced transformation and modernization, that economic growth was reflected in the change and improvement in the QOL of inhabitants (Gomułka, 2016). Unfortunately, the economy was still afflicted with a relatively high level of unemployment, which encouraged labour migration of Poles to Western Europe (Kindler, 2018). On the path of economic growth in Poland, a significant turning point was the accession to the EU, which brought the opening of the economy and an increase in the impact of external determinants (European integration, globalization, modernization, metropolitanization). This resulted in many positive and negative consequences. On the one hand, economic growth and diffusion of civilizational progress in subsequent years contributed to the achievement of a higher level of satisfaction of needs, while these needs were also changing and new ones were also developing. On the other hand, accession to the EU, by opening borders and allowing free movement of persons, goods and capital, led to an increase in the outflow of workers, which brought about negative social consequences (Belka, 2013). As a result, economic growth did not lead to improvement in the QOL of Poles and thus did not immediately result in socio-economic development.

**Fig. 1:**
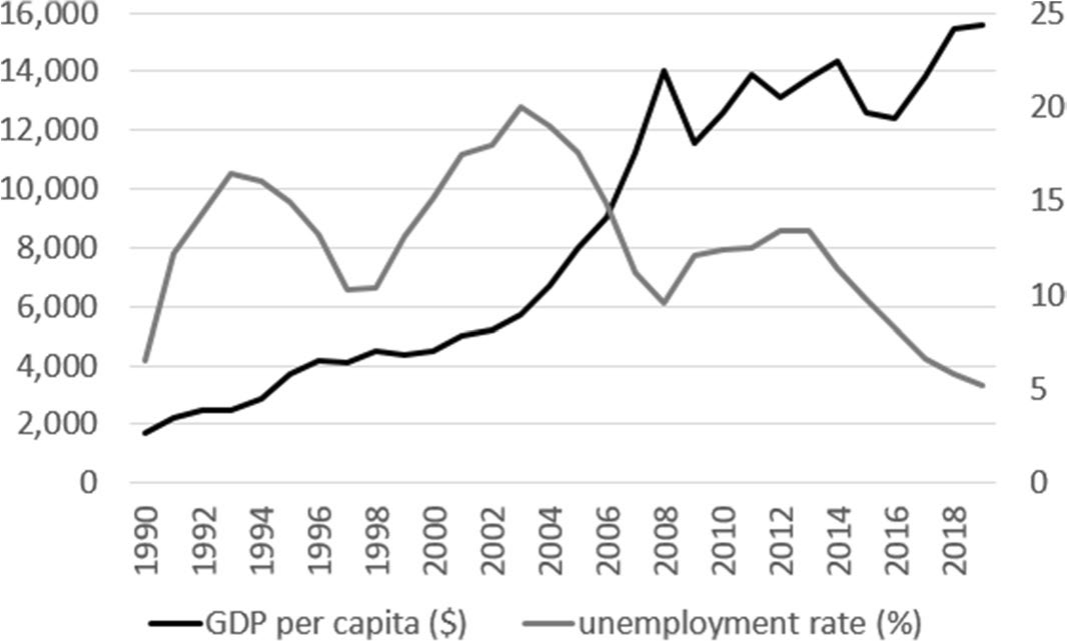
GDP per capita and the unemployment rate in Poland in 1990–2019. Source: own elaboration

Overall, relationships between the rate of economic growth and QOL in Poland in the period 1990–2018 can be determined on the basis of the gross domestic product (GDP) curve as a measurement of quantitative economic growth and the curve of the unemployment rate–an important negative indicator of QOL (Fig. 1). Two phases can be distinguished in the development of these relationships, covering the years 1990–2008 and 2008–2019. In the first phase, with a relatively stable increase in GDP per capita, alternating increases and decreases in the unemployment rate appeared (during the period of systemic transformation). At the end of the first phase, upon accession to the EU, Poland’s economy had unfavourable decreasing trends in economic growth rates and very high unemployment. It was only in the second phase, when the pace of economic growth was stable, that there was a visible, regular fall in unemployment (from 15 to 5%), which is indicative of a noticeable improvement in QOL.

**Fig. 2:**
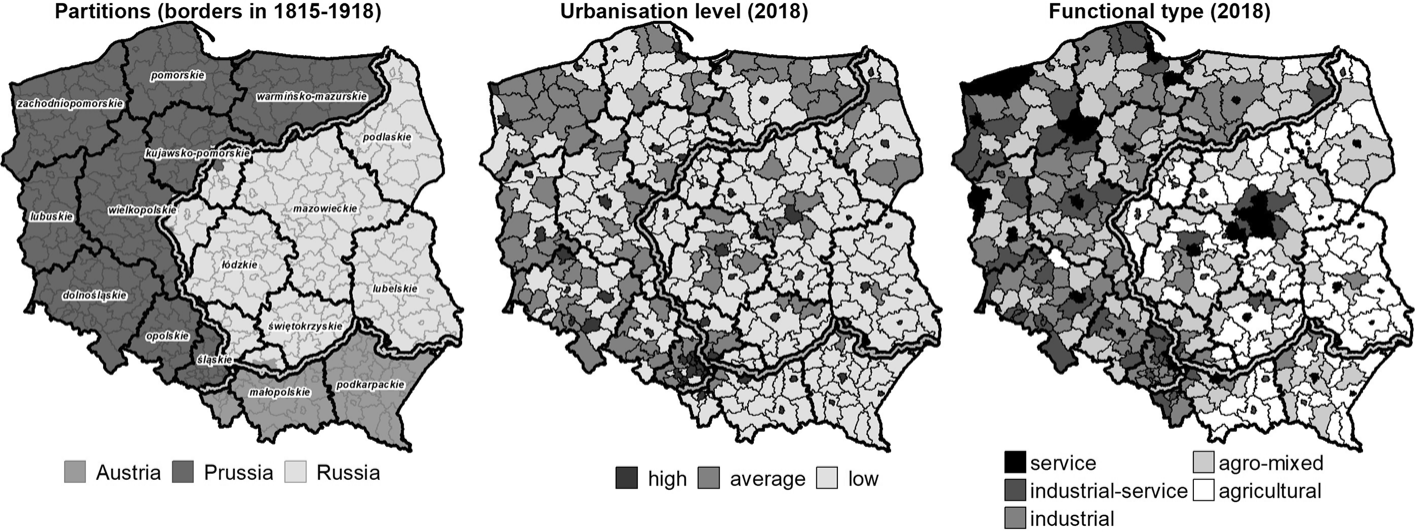
Determinants of spatial differences in socio-economic development in Poland. Source: own elaboration

The spatial structure of the socio-economic development level in Poland, including QOL, is characterized by large, well-established differences. These are marked: (1) between the western and eastern part of Poland, (2) on an interregional scale and (3) on a regional scale, between the main city and rural areas. Spatial differences in development are connected to the historical legacy dating back to the period of the Prussian, Russian and Austrian partitions of Polish land (1795–1918; Grosfeld & Zhuravskaya, 2015). The partitions varied in terms of urbanization level and the degree of diversification in economic structure (Fig. 2). Lack of empowerment, coinciding with a period of intensive industrialization and socio-economic changes, left a deep mark on the socio-economic structure of Poland. After World War II, the centralized management and localization policy typical of a planned economy exacerbated the differences in spatial development during communism. Also, at the beginning of systemic transformation (i.e. since 1990), the economic recession and difficulties in the adaptation to the new economic system–particularly in industry and agriculture–strengthened spatial developmental inequalities. Therefore, to a large extent this approach refers to historical institutionalism, highlighting the persistence of institutions and their role in shaping the contemporary societies and economies in specific territories (Pierson, 2004; Shirley, 2005; Thelen, 2004)).

In this cognitive situation, what seems justified is an attempt to answer the following questions: Was the contemporary socio-economic development phase of Poland (dated from 2004) a period of social progress and an increase in QOL, and if so, to what extent? What are the spatial regularities in the progress in QOL at the local level? The analysis of QOL is here discussed in geographical terms. Because of strong spatial diversities in QOL in Poland, counties (poviats, Polish: *powiaty*)–that is, the second-tier territorial units in the country’s administrative division–which are part of 16 voivodeships (regions) in Poland, are used as a system of reference. The application of the pattern of counties, hereafter called a local pattern, provides a higher degree of spatial detail in the research compared to regional patterns and makes it possible to recognize intraregional differences.

It seems undisputable that, in Poland after 2004, there was a major increase in the importance of QOL and social progress. This is a phase of socio-economic development, positively oriented to social changes, characterized by a sequence of social changes “pushed” by domestic and regional policy. The socio-economic changes in question are highly complex and spatially diversified, demonstrating a significant relationship to the consequences of being part of the EU.

The present analysis is based on the operational definition of QOL. It has been assumed that the structural components of QOL are groups of needs, related to the basic spheres of human’s existence. Based on the output in the field of social pre-theory in human geography (Knox, 1973; Pacione, 1982, 1986; Smith, 1977) and with reference to the classification used in the regional statistics of the OECD QOL (2018), what is distinguished are 11 constituents describing the level of QOL, grouped in two dimensions. The first dimension consists of material constituents including income level, job availability and housing. The second dimension comprises social constituents of QOL which result from the state of health, availability of education, environmental conditions for life, the security level, a degree of social engagement and access to services (Table 1).

**Table 1:** Model of the life quality structure. Source: own elaboration

Dimensions		Constituents	Indicators (measurement unit) [available years]
Overall (O)	Material dimension (M)	Income and wealth	IW1: commune and county incomes from PIT per capita (złoty/person) [2004, 2018] IW2: average remuneration (złoty) [2004, 2018]
		Work	WK1: unemployment rate (%) [2004, 2018] WK2: share of unemployed with higher education in working age population number (%) [2004, 2018]
		Housing	HG1: share of population using sewer system (%) [2004, 2018] HG2: average housing area (m^2^) [2004, 2018]
	Social dimension (S)	Health	HH1: tumour deaths (person/100,000 inhabitants) [2004, 2018] HH2: cardiovascular disease deaths (person/100,000 inhabitants) [2004, 2018]
		Education	EN1: foreign language learners (person/100 inhabitants aged 7–19) [2008, 2018] EN2: proportion of children in pre-school education (%) [2004, 2018]
		Environment	ET1: emission of dust and gas pollutants (ton/year) [average from 2004–2006 and 2016–2018] ET2: population number per 1 ha of green areas (person/ha) [2004, 2018]
		Safety	SY1: total crimes found by police (number of crimes/1,000 inhabitants) [2012, 2018] SY2: average number of evictions from apartments (number of evictions/10,000 inhabitants) [average from 2003–2010 and 2011–2018]
		Civil engagement	CE1: turnout in local elections (vote on heads of rural commune governments, mayors-first run-off) (%) [2006, 2018] CE2: number of sports club members (person/1,000 inhabitants) [2004, 2018]
		Availability of services	AS1: average number of doctors (physician/10,000 inhabitants) [2006, 2018] AS2: average number of hypermarkets and supermarkets (market/100,000 inhabitants) [2008, 2018]

## Data and Methods

The empirical study of QOL in Poland concerns conditions in 2004 and 2018, which is the time frame for the contemporary development phase. The comparison of these years constitutes the basis for the analysis of changes. The spatial pattern of the research is a local system (LAU1–counties) composed of 380 units. It has been assumed that the model of a QOL structure is constant for the years in question (i.e. the constituents and indicators describing them are constant). The choice of constituents and indicators was based on the assumption that, in the years studied, they were the main vehicles of change in QOL and was prepared on the basis of available statistical data. The data are contained in two geographical matrices of information in the form of the overall (O) QOL (380 counties and 18 indicators) for 2004 and 2018 in which submatrices are distinguished related to two subsets of constituents and their indicators: (M) the material dimension (380 × 6) and (S) the social dimension (380 × 12). The indicators, adopted with the analysis in mind, certainly fail to exhaust the description of the discussed dimensions due to their possibly wide interpretation as well as the fact that they pertain chiefly to the quantitative aspect, disregarding the qualitative aspects (e.g. the number of physicians rather than the quality of healthcare, the income rather than satisfaction with life). Nevertheless, they can be deemed relatively suitable for describing the dimensions in question because they allow to describe, in a satisfying way, the specific QOL differentiators of importance to the effects of Poland’s social and economic transformation and the results of accessing the EU which can be viewed as a big leap in development. What is more, research suggests that elements like high income (Pittau et al., 2009; Seghieri et al., 2006), stable employment (Bouazzaoui & Mullet, 2002) and good health (Mroczek & Spiro, 2005) show a positive correlation with wellbeing. Just like the other elements related to enhancing living conditions (housing, educations, environment, safety–Böhnke, 2007, Somarriba & Pena, 2009, Ivaldi et al., 2016) which are of importance specifically in countries in the process of transformation and modernisation. Nevertheless, Poland lacks many comparable and credible data that allow to provide QOL indicators (specifically in a broad approach). In general, the problem is in the irregularity of access to data, poor frequency, a random nature of the phenomena at hand, the ambiguity accompanying the definition of the indicators and their variability over time, the different levels of spatial units and the ambiguity of the population (more on these issues in Michalski, 2020). Therefore, the indicators adopted in the article are a compromise between the availability of data and our research intent. In our opinion, they represent a basis sufficient for carrying out a QOL analysis in a narrow approach.

The first step in the analytical procedure was the reduction of the indicators describing the constituents of QOL in the matrices of geographical information by means of principal component analysis (PCA; Morrison, 1967). The legitimacy of the PCA application was determined by the Bartlett test χ^2^ and the Kaiser–Mayer–Olkin coefficient (Vehkalahti & Everitt, 2019). As a result of the PCA procedure, principal components were obtained constituting the linear combination of original indicators. For the overall QOL as well as the material dimension and social dimensions for both investigated periods (2004 and 2018), the first principal component explaining the largest proportion of the variance of the original indicators was selected for further analysis. The interpretation of the principal components was based on the values of the linear correlation coefficients between principal components and the original indicators (and de facto constituents of QOL).

Second, the spatial distribution of the values of the first principal components for overall QOL (PCA_04_O, PCA_18_O) as well as the material (PCA_04_M, PCA_18_M) and social dimensions (PCA_04_S, PCA_18_S) was analysed. This step constitutes the basis for the spatial analysis of QOL in the investigated dimensions in the two time periods. Because the values of the principal components are standardized variables, there is a limited possibility of comparing their values across years. To analyse changes in QOL, we used differences in the ranks of local units organized by descending values of principal components. The difference of ranks can be treated as a change in the position of local units at the scale of QOL–that is, a relative increase or decrease in QOL. Changes in the position of local units on the QOL scale by + / − 10 were treated as a stable situation, changes within + / − 11–30 as an increase/decrease in QOL and changes above + / − 30 as a significant increase/decrease in QOL. It should be emphasized, however, that these changes are relative, which means that an increase in QOL in some counties results in a decrease in others. Thus, this situation does not necessarily mean a real decrease in the units characterized by a decrease in the position on the QOL scale, and can only result from a larger increase in others. In the analysis of the spatial distribution of QOL, each value of the first principal components and differences in ranks (a measurement of changes in QOL) were tested for the existence of spatial autocorrelation (Anselin, 1988, 1995). The occurrence of statistically significant clusters of counties, similar in terms of QOL, was the focus of investigation. To this end, use was made of Moran’s I statistics (global and local).

Third, the analysis of the spatial differences in QOL was complemented by the analysis of the degree of inequality and the direction of these changes. For this purpose, use was made of a classic measurement–the Gini index^2^ (Dixon et al., 1987).

In the last, fourth step, we checked whether the distinguished historical (former political-administrative divisions of partitions) and contemporary (urbanization level and functional types) determinants of spatial differences in socio-economic development played a significant role in the spatial differences for QOL in 2004 and 2018. Because the specified principal components do not have normal distribution, we used Kruskal–Wallis one-way analysis of variance by ranks (Kruskal–Wallis ANOVA; Kruskal, 1952; Kruskal & Wallis, 1952). To indicate precisely which groups are significantly statistically different, the post-hoc Dunn test was applied with correction for tied ranks, with Bonferroni correction because of multiple testing (Zar, 2014). To check if QOL in the studied groups of local units changed with statistical significance between 2004 and 2018, the Wilcoxon matched-pairs test was used (Wilcoxon, 1945, 1949).

## Results

As a result of the PCA, the first principal components for each of the three analysed dimensions of QOL in 2004 and 2018 were separated (Table 2). Both the Bartlett test and the values of the KMO coefficient indicate the legitimacy of the PCA application. Because the principal components show a statistically significant correlation with indicators describing all constituents of QOL, the components are interpreted in line with the dimensions (overall, material, social) and treated as their synthetic indicators. On the basis of changes in the values of correlation coefficients between 2004 and 2018, the role of the indicators describing tumour deaths (health), unemployment rate (work) and level of crime and evictions (safety) in the structure of the principal components clearly increased, while the role of cardiovascular disease deaths (health), turnout in local elections (civil engagement) and the share of the unemployed with higher education (work) markedly decreased. Thus, all of the highlighted components of QOL are adequately represented in the principal components. The percentage of the explained variance of the first principal components amounts to 33% and 30% in the overall QOL, in the material dimension 47% and 44% and 31% and 27% in the social dimension for 2004 and 2018, respectively.

**Table 2:** Typical features of the first principal components. Source: own elaboration

	Overall (O)		Material dimension (M)		Social dimension (S)	
	PCA_04_O	PCA_18_O	PCA_04_M	PCA_18_M	PCA_04_S	PCA_18_S
Bartlett test ${\chi }^{2}$	3461.58	3248.36	1047.88	824.83	1155.84	1011.84
df	153	153	15	15	66	66
p-value	< 0.000001	< 0.000001	< 0.000001	< 0.000001	< 0.000001	< 0.000001
KMO (Keiser–Mayer–Olkin)	0.828	0.759	0.66	0.638	0.805	0.726

% of variance		33.16		29.556		47.409		44.012		30.833		27.122	
Linear correlation coefficients of principal component with original indicators	IW1_04/18	− 0.781	****	− 0.744	****	− 0.846	****	− 0.870	****				
	IW2_04/18	− 0.548	****	− 0.603	****	− 0.715	****	− 0.760	****				
	WK1_04/18	0.243	****	0.453	****	0.276	****	0.647	****				
	WK2_04/18	− 0.368	****	0.100	ns	− 0.475	****	0.267	****				
	HG1_04/18	− 0.910	****	− 0.844	****	− 0.855	****	− 0.772	****				
	HG2_04/18	0.758	****	0.676	****	0.764	****	0.468	****				
	HH1_04/18	− 0.194	***	− 0.491	****					− 0.144	**	- 0.561	****
	HH2_04/18	0.387	****	0.160	**					0.427	****	0.057	ns
	EN1_04/18	0.069	ns	− 0.040	ns					0.112	*	− 0.053	ns
	EN2_04/18	− 0.804	****	− 0.726	****					− 0.810	****	− 0.662	****
	ET1_04/18	− 0.280	****	− 0.240	****					− 0.235	****	− 0.187	***
	ET2_04/18	0.474	****	0.473	****					0.544	****	0.515	****
	SY1_04/18	− 0.417	****	− 0.609	****					− 0.466	****	− 0.666	****
	SY2_04/18	− 0.680	****	− 0.707	****					− 0.700	****	− 0.752	****
	CE1_04/18	0.686	****	0.220	****					0.752	****	0.342	****
	CE2_04/18	− 0.181	***	0.053	ns					− 0.231	****	0.021	ns
	AS1_04/18	− 0.792	****	− 0.749	****					− 0.739	****	− 0.715	****
	AS2_04/18	− 0.743	****	− 0.680	****					− 0.778	****	− 0.747	****

ns – not significant

****p ≤ 0.0001; ***p ≤ 0.001; **p ≤ 0.01; *p ≤ 0.05

**Fig. 3:**
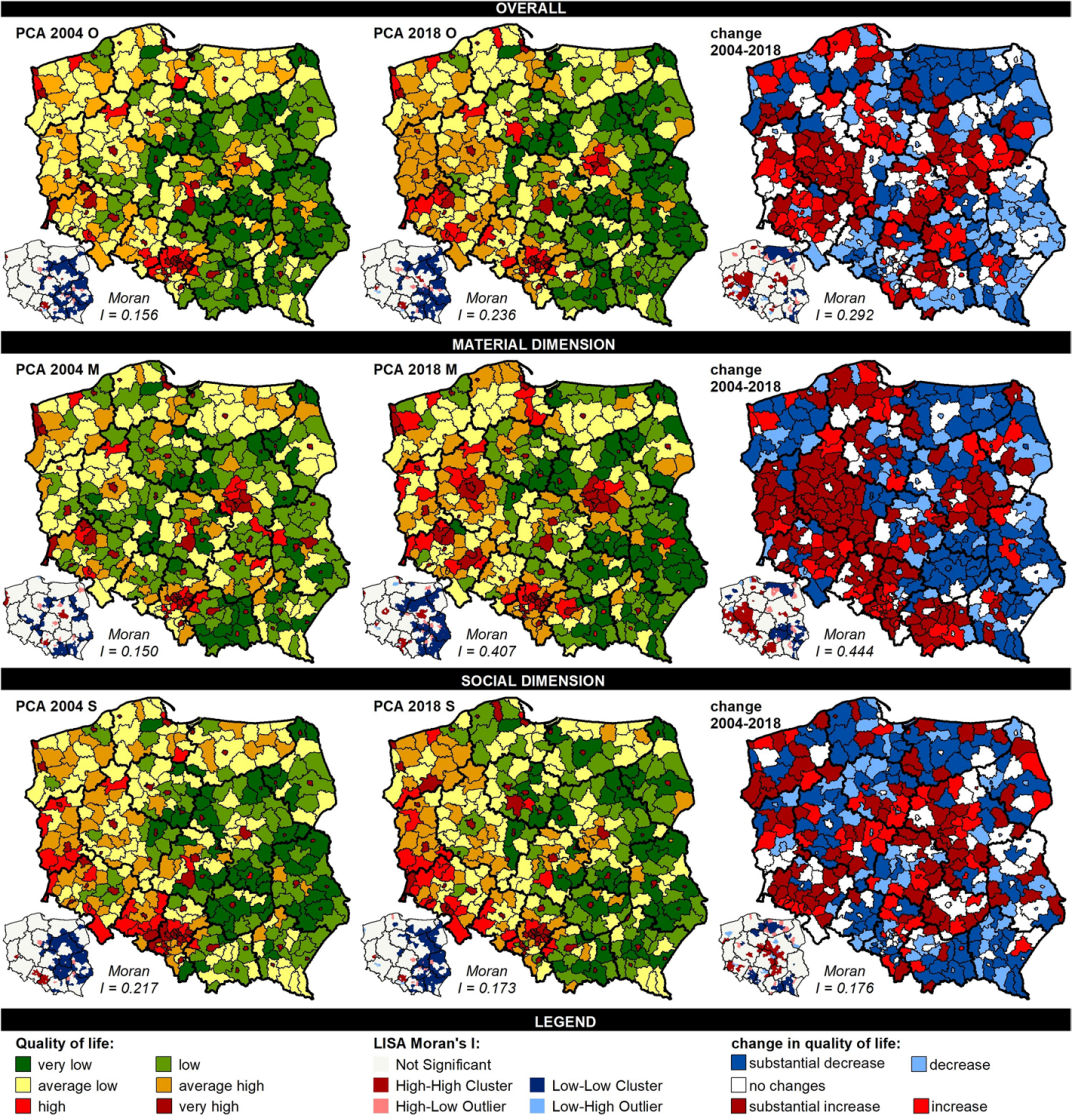
Spatial differences in quality of life in Poland at the local level. Source: own elaboration

In 2004 and 2018, very high and high overall QOL was observed mainly in highly urbanized counties. These are primarily the largest cities in terms of population, which are relatively evenly distributed across the country (Fig. 3). In 2004 and 2018, these counties made up 22% and 24% of all investigated units, respectively, populated by 38% and 40% of the country’s residents. Counties with an average (average high and average low) overall QOL constituted the most numerous group of units. In 2004, their share was 42%, but they were populated by 35% of the country’s inhabitants, and in 2018 it was 44% and 36%, respectively. These types of counties dominated in the western part of Poland and appear sporadically in the eastern part, mainly around the largest cities. Meanwhile, the share of counties with low and very low overall QOL was reduced from 35 to 32%; therefore, the proportion of the population living in such areas decreased from 27 to 24%. There is a clear concentration of counties with the lowest overall QOL in the eastern part of the country. Clusters of counties with high QOL were relatively small and formed around the largest cities. Very large clusters of low QOL were created by counties in the eastern part of the country, which make a clear reference to the historically determined low level of urbanization and agricultural functional types. The biggest positive changes in the overall QOL occurred primarily in western Poland (especially in Wielkopolskie Voivodeship) and in the eastern and southern part of the country, mainly around the largest cities–Warsaw, Krakow and Łódź–that is, in areas already characterized by a relatively high QOL. The largest cluster of counties with unfavourable changes on the QOL scale was observed in the eastern part (and exceptionally in Zachodniopomorskie Voivodeship). These are primarily agricultural or agro-mixed areas, with a low level of urbanization; their development level is low and they are often marked (e.g. in Zachodniopomorskie and Warmińsko-Mazurskie Voivodeships) by structural unemployment related to the liquidation of state agricultural farms at the outset of the systemic transformation.

It is worth noting that greater changes in QOL occurred in the material dimension than in the social dimension. Regarding the material dimension, the proportion of counties with high and average QOL increased from 24 to 26% and from 38 to 42%, respectively. This caused the percentage of people living in counties with a high material QOL to increase from 40 to 42%, and with an average QOL, from 32 to 35%. Meanwhile, the share of counties with low material QOL was reduced from 38 to 33%, and the proportion of the population living in such areas decreased from 28 to 23%. The spatial regularities for the material dimension are nearly the same as for overall QOL. However, a clearly higher material QOL can be found in highly urbanized counties with a prevalence of services and industry in the economic structure. Additionally, counties neighbouring these counties show noticeably higher positive changes in the material dimension of QOL. The most positive changes are particularly visible in the western part of the country (Wielkopolskie, Dolnośląskie, Lubuskie, Opolskie, Pomorskie and Śląskie Voivodeships) and around Warsaw and Krakow. On the other hand, large clusters of counties with low material QOL can be clearly noticed in the eastern part of Poland (which expressly refers to historical patterns – the Russian and Austrian partitions).

In overall QOL and in the material dimension, there was a clear increase in the share of counties and their population with high and average QOL, but the situation in the social dimension looked somewhat different. In the social dimension, high QOL can be observed in about 25% of counties and 40% of the population during the investigated years. In the case of average QOL, the proportion of counties decreased from 44 to 43%, but the share of the population was reduced from 36 to 33%. What occurred, however, was an increase in the share of counties with low social QOL from 31 to 32%, and, what is worse, there was a significant growth in the share of the population living in these types of counties, from 24 to 27%. Counties with a high and average social QOL dominate in the western (especially in the south-western) part of the country, and those with the lowest social QOL are in the eastern part of the country. This is also indicative of the great importance of historical determinants. The spatial differences in QOL in Poland at the local level are, to a large extent, permanent, and this is related to (1) the historical legacy dating back to the partitions, (2) the level of urbanization and (3) the economic structure. Hence, in the further stages of this research, these aspects are taken into account in the analysis of spatial differences in QOL.

Despite the historically established and persistent spatial differences in QOL, there is visible tendency for inequalities to decline. This has been confirmed in the decreasing values of the Gini index (Table 3). The greatest inequalities at the national level–but also their greatest reduction–were observed in the material dimension of QOL, while the smallest differences can be found in the social dimension. In overall QOL, the greatest differences exist in the counties of eastern Poland, mainly those within the borders of the former Russian and Austrian partitions, with a low urbanization level and those of agricultural and industrial types. In contrast, the smallest differences in QOL can be found in the counties of the former Prussian partition, those with an average and high urbanization level and also the agro-mixed types. However, while there has been a decrease in inequality in most cases, there has also been an increase in inequality in the group of counties with a high urbanization level and dominated by services, and also in the group of agricultural counties with a low level of urbanization. The tendencies are similar in the material dimension–in general, differences are decreasing. It should be noted that a decrease in inequality in the material dimension in the counties of the former Russian and Austrian partitions dropped in the years 2004–2018 to the level of inequality recorded in 2004 in the counties of the former Prussian partition. Inequalities in this dimension decreased the most in industrial and agro-mixed counties. Meanwhile, high differences persist in counties with a low level of urbanization and of an agricultural type, and in service counties there has been a marked increase in inequality. Against this background, social inequalities in QOL are the smallest. Admittedly, inequalities in this dimension are at an all-time high in the counties of the former Russian and Austrian partitions, especially in comparison to the counties of the Prussian partition; still, in the other aspects, these inequalities are smaller. It is worth noting that the level of the reduction in inequalities in the social dimension is significantly lower and, in some cases, a minor increase has been recorded (e.g. in counties with a high urbanization level or service and agricultural types).

**Table 3:** Level of inequalities in terms of quality of life (values of the Gini index). Source: own elaboration

Aspect		Overall (o)		Material dimension (m)		Social dimension (s)	
		PCA_04_O	PCA_18_O	PCA_04_M	PCA_18_M	PCA_04_S	PCA_18_S
POLAND		0.254	0.231	0.310	0.222	0.218	0.187
Partition	Austria	0.290	0.282	0.373	0.271	0.240	0.234
	Prussia	0.201	0.177	0.277	0.179	0.163	0.153
	Russia	0.310	0.282	0.350	0.264	0.271	0.217
Urbanization level	High	0.097	0.102	0.137	0.137	0.099	0.103
	Average	0.110	0.108	0.162	0.142	0.108	0.098
	Low	0.170	0.184	0.226	0.200	0.166	0.144
Functional type	Service	0.146	0.157	0.190	0.213	0.142	0.143
	Industrial-service	0.189	0.167	0.216	0.159	0.182	0.169
	Industrial	0.152	0.136	0.213	0.131	0.134	0.128
	Agro-mixed	0.136	0.116	0.214	0.123	0.123	0.117
	Agricultural	0.194	0.218	0.261	0.194	0.184	0.187

The results of Kruskal–Wallis ANOVA and post-hoc tests (Table 4) and the Wilcoxon matched-pairs test (Table 5) confirmed, in the vast majority of cases, the existence of statistically significant differences in QOL and its changes among counties located within the borders of the former partitions, which are distinct in terms of urbanization level and economic structure.

**Table 4:** Results of Kruskal–Wallis ANOVA for the distinguished aspects of spatial differences. Source: own elaboration

aspect	Quality of life:		Overall			Material dimension			Social dimension		
	year		2004	2018	change*	2004	2018	change*	2004	2018	change*
Partitions	Median of group	Austria	− 1.366	− 1.380	− 12.000	− 0.956	− 0.748	− 4.000	− 0.628	− 0.941	− 22.000
		Prussia	− 0.053	0.243	2.000	− 0.216	0.251	15.000	0.246	0.119	− 5.000
		Russia	− 1.560	− 1.290	− 2.000	− 0.769	− 0.911	− 19.500	− 1.368	− 0.868	10.500
	H** (df = 2)		48,287	48.287	54.628	6.202	12.875	46.830	33.986	70.928	52.275
	*p*-value (α = 0.05)		< 0,000,001	< 0.000001	< 0.000001	0.045	0.002	< 0.000001	< 0.000001	< 0.000001	< 0.000001
Urbanization	Median of group	Low	− 1.588	− 1.350	− 0.500	− 1.085	− 0.914	6.000	− 1.107	− 0.943	1.000
		Average	0.419	0.399	− 1.500	0.044	0.208	− 2.500	0.247	0.282	3.500
		High	4.160	3.832	0.000	2.644	2.001	− 4.000	3.158	2.764	− 2.000
	H** (df = 2)		270.222	270.222	241.455	4.468	247.186	181.688	8.956	244.778	236.896
	*p*-value (α = 0.05)		< 0,000,001	< 0.000001	< 0.000001	0.107	< 0.000001	< 0.000001	0.011	< 0.000001	< 0.000001
Functional type	Median of group	Service	4.803	3.828	− 1.000	2.987	2.025	− 5.000	3.385	2.681	− 1.000
		Industrial-service	1.411	2.154	3.000	1.304	1.256	1.000	1.303	1.799	5.000
		Industrial	0.066	0.266	3.500	0.011	0.559	26.500	0.256	− 0.050	− 21.500
		Agro-mixed	− 1.056	− 0.944	− 4.000	− 0.886	− 0.606	− 8.000	− 0.644	− 0.629	5.000
		Agricultural	− 2.385	− 2.368	− 7.000	− 1.291	− 1.707	− 17.000	− 1.998	− 1.697	4.000
	H** (df = 4)		271.133	271.133	280.269	4.258	210.148	269.782	30.680	259.362	224.376
	*p*-value (α = 0.05)		< 0.000001	< 0.000001	< 0.000001	0.372	< 0.000001	< 0.000001	0.000	< 0.000001	< 0.000001

*Change–expressed as a difference of ranks between 2004 and 2018 on the scale of quality of life

**H–values of H statistics with correction for tied ranks

**Table 5:** Results of the Wilcoxon matched-pairs test for PCA values in 2004 and 2018. Source: own elaboration

(A) Partitions	Austria (49)			Prussia (185)			Russia (146)		
	O	M	S	O	M	S	O	M	S
median of differences	0.242	0.027	0.422	− 0.103	− 0.323	0.144	0.078	0.330	− 0.277
Σ of ranks +	403	559	302	10,082	12,218	7572	4831	2913	7005
Σ of ranks −	822	666	923	7123	4987	9633	5900	7818	3726
T	403	559	302	7123	4987	7572	4831	2913	3726
*p*-value	0.037	0.601	0.002	0.042	0.000	0.158	0.298	0.000	0.001
Z	2.079	0.527	3.084	2.028	4.957	1.412	1.043	4.790	3.202
*p*-value	0.038	0.598	0.002	0.043	0.000	0.158	0.297	0.000	0.001

(B) Urbanization level	High (70)			Average (90)			Low (207)		
	O	M	S	O	M	S	O	M	S
median of differences	0.460	0.556	0.421	− 0.036	− 0.159	− 0.063	− 0.071	− 0.096	− 0.111
Σ of ranks +	461	464	664	2110	2281	2084	13,136	12,666	12,272
Σ of ranks −	2024	2021	1821	1985	1814	2011	8392	8862	9256
T	461	464	664	1985	1814	2011	8392	8862	9256
*p*-value	0.000	0.000	0.001	0.804	0.350	0.885	0.006	0.027	0.081
Z	4.571	4.553	3.383	0.249	0.938	0.145	2.748	2.204	1.747
*p*-value	0.000	0.000	0.001	0.803	0.348	0.885	0.006	0.028	0.081

(C) Functional type	Service (31)			Industrial-service (47)			Industrial (54)			Agro-mixed (87)			Agricultural (74)		
	O	M	S	O	M	S	O	M	S	O	M	S	O	M	S
median of differences	0.760	0.810	0.534	0.053	0.079	0.031	− 0.201	− 0.669	0.239	− 0.102	− 0.126	− 0.093	0.064	0.239	− 0.348
Σ of ranks +	59	45	106	499	458	552	946	1326	449	2389	2258	2199	1235	739	1863
Σ of ranks −	437	451	390	629	670	576	539	159	1036	1439	1570	1629	1540	2036	912
T	59	45	106	499	458	552	539	159	449	1439	1570	1629	1235	739	912
*p*-value	0.000	0.000	0.004	0.498	0.267	0.904	0.080	0.000	0.011	0.044	0.147	0.230	0.415	0.000	0.010
Z	3.694	3.968	2.773	0.683	1.116	0.122	1.748	5.020	2.523	2.008	1.454	1.204	0.819	3.491	2.559
*p*-value	0.000	0.000	0.006	0.495	0.264	0.903	0.080	0.000	0.012	0.045	0.146	0.229	0.413	0.000	0.010

O–overall, M–material dimension, S–social dimension; the number of units in a given group is provided in brackets

In overall QOL, there are statistically significant differences between the counties situated in the former Prussian and Austrian partitions and in the Prussian and Russian ones (in 2004 and 2018, and for the change in QOL). A lack of differences is only visible between the counties of the former Austrian and Russian partitions. The highest QOL and its greatest positive changes were observed in the counties located in the former Prussian partition. Meanwhile, a slight decrease in QOL was recorded in the counties of the former Austrian partition. In the material and social dimensions of QOL (in both investigated years and for changes in QOL) statistically significant differences were identified between the counties of all former partitions. The counties of the former Prussian partition were characterized by the highest QOL in these dimensions. In the investigated period, the greatest positive changes in the material dimension occurred in the counties of the former Prussian partition, and the most considerable negative ones took place in the counties of the former Russian partition. In contrast, in the social dimension, the greatest increase in QOL occurred in the counties of the former Russian partition, and a slight decrease was noted in the counties of the former Austrian partition.

Counties with a high, average and low levels of urbanization in 2004 and 2018 are statistically significantly different in terms of overall QOL and in both the material and social dimensions. These types of differences do not exist in the case of changes in overall QOL and changes in the social dimension of QOL. Only in the material dimension of QOL did statistically significant differences exist between counties with low and average as well as low and high urbanization levels. The highest QOL was observed in the counties with the highest urbanization level, and the lowest was seen in the counties with the lowest level of urbanization. Although the greatest positive changes in the material dimension of QOL occurred in the counties with a low urbanization level, the smallest (a relative reduction in this regard) in the counties with a high urbanization level.

There are also statistically significant differences in overall QOL between all functional types of counties in both investigated years, although there is a lack of differences regarding *changes* in overall QOL. Nonetheless, what is observable is a relative decrease in overall QOL in service counties and an increase in industrial and agro-mixed ones. However, in the material and social dimensions of QOL, the lack of statistically significant differences is observed only between QOL in counties representing service as well as industrial-service types–only in 2018. In the case of changes in material QOL, significant differences were recorded between industrial-service and service counties; industrial and agricultural ones; industrial and agro-mixed as well as service counties; and between those of agro-mixed and agricultural types. The most considerable positive changes in QOL occurred in industrial counties, while service and agricultural counties experienced the greatest relative negative changes. A lack of statistically significant changes in social QOL was noticed in industrial-service counties with agricultural ones; agro-mixed with service and agricultural counties; and agricultural with service counties. At the same time, a decrease in social QOL was observed in service and industrial counties, while agricultural ones saw an increase in this respect.

## Discussion

Despite declining inequalities, changes in QOL in the years 2004–2018 heighten contrasts between western and eastern Poland. A marked increase in overall QOL occurred mainly in the counties of western voivodeships: Wielkopolskie and Dolnośląskie, and also in Mazowieckie, which were characterized by the highest economic growth (see classification of the EU 2021–2027 Cohesion policyThis corresponds with the regularities indicated in the results of research into regional differences in the quality of life in Poland, carried out by Sompolska-Rzechuła (2017). Moreover, this situation corroborates the results of research conducted by Roszko-Wójtowicz and Grzelak (2018) into the quality of life in Wielkopolska and in Masovia as well as research carried out by Dudek et al. (2016) for Lower Silesia. In the material dimension, an improvement in QOL was observed in almost all of western Poland, except for Zachodniopomorskie Voivodeship (with former state agricultural farms), and in Małopolskie and Mazowieckie Voivodeships (impact zones of Krakow and Warsaw). The largest group of counties with the highest increase in QOL can be found in the western part of Wielkopolskie Voivodeship. Meanwhile, in the social dimension, a relatively high increase was noted in many counties dispersed throughout the country and in two spatially compact patterns of counties in central Poland (the borderlands of Mazowieckie, Łódzkie and Kujawsko-Pomorskie Voivodeships) and in Dolnośląskie Voivodeship. A relative decline in overall QOL was recorded in the counties of Warmińsko-Mazurskie Voivodeship in the north-eastern part of the country (on the border with Russia). Some counties of Świętokrzyskie and Lubelskie Voivodeships, however, saw a fall only in material QOL, whereas the counties of Opolskie and Podkarpackie Voivodeship saw declines in the social dimension. In general, counties with a deterioration in QOL are characterized by a lower level of economic growth. Nevertheless, the processes taking place lead to a reduction in the scale of inequalities. However, greater inequalities concern the material dimension more than the social one, and it was in the case of the material dimension that a greater decrease in inequalities was observed. This confirms the possibility of closing gaps in terms of the material QOL with relative ease and speed by public intervention (e.g. creation of new jobs, minimum wage increases), while improvements are not that simple in the social dimension, especially in a period of transformation and major development shocks (Bidzan-Bluma et al., 2020).

Spatial differences in QOL in contemporary Poland are strongly influenced by historical determinants resulting from more than 120 years of lost independence and the fact that its present-day territories belonged to the three completely different economic and political systems of Prussia, Russia and Austria (Bukowski & Novokmet, 2019). The historical background is one of the main reasons for the differences in the current development situation of individual territories. Political divisions and their consequences connected with functioning in different political, economic and social conditions, in line with the principle of path dependence, become deep-seated determinants of developmental processes, stressing the fact that “history matters” (Boas, 2007; Peters et al., 2005). The counties situated in the former Prussian partition are observed to have a higher QOL than those in the former Russian and Austrian partitions. It should be remembered, however, that partitions strengthened rather than created the historical determinants of today’s spatial differences in QOL. As Hryniewicz (2003, p. 77) puts it: *Until 1795, Poland’s economic growth was characterized by fairly large territorial differences, with a clear developmental predominance of Wielkopolska (Wielkopolskie Voivodeship). Differences in economic growth had cultural and structural bases. The partition borders overlapped with the pre-existing differences of economic levels and the partition era did not significantly change the disparities in the development of Polish land. The analysis of modern development tendencies proves that the differences in economic growth outlined several hundred years ago persist to date. The determinative role of long-duration processes consists, in particular, in drawing a clear distinction between the western part of the country, more developed, and eastern—less developed*. Those differences correspond to the general patterns of spatial differences in contemporary Poland (Zarycki, 2007), which were consolidated in the period of more than four decades under a socialist economy (Dyba et al., 2018). The areas of the former Prussian partition are additionally characterized by the largest decreases in inequalities in the overall and material dimensions of QOL. This confirms the relatively more favourable development opportunities of former Prussian partitions, resulting in higher QOL, emphasized by, for example, the research results of Bukowski et al. (2017). Counties of the former Russian and Austrian partitions are much more internally diversified than counties of the ex-Prussian partition. These differences are marked throughout the entire analysed period and concern all three considered dimensions of QOL; this has been confirmed by changes in QOL in the investigated period. The counties of the former Prussian partition, with a higher level of economic growth, including a higher level of remuneration and a lower unemployment rate, have seen an increase in the overall QOL. Meanwhile, in the economically weaker counties of the previous Austrian partition, a decrease in QOL has been observed; in the counties of the former Russian partition, diversified in terms of economic development level, no significant differences have been identified. In the social dimension, an improvement in QOL has been noticed in the counties of the former Russian partition, which was characterized by the most serious deficits in this respect. A fall in social QOL was recorded in the areas of the former Austrian partition, which was the most internally diversified in this dimension.

The second aspect discussed here that determined spatial differences in QOL is the level of urbanization. The impact of urbanisation on the changes to the QOL has been largely discussed in literature on the subject (Bhattarai & Budd, 2019; Kuddus et al., 2020; Rameli et al., 2020). The obtained results indicate that counties with a higher level of urbanization, which triggers or accelerates the process of socio-economic change, are observed to have higher QOL than counties with an average and low urbanization level. The highest QOL can be found in the functional areas of the main regional centres making up a polycentric network of growth poles and socio-economic growth (Churski, 2014). This is particularly visible in material QOL, which strongly indicates the efficiency of urbanized areas in creating diversified and buoyant labour markets, strengthened by the effective absorption of European funds (Churski et al., 2016). Counties with the lowest levels of urbanization are characterized by the most substantial discrepancies in QOL in each year considered and in each of the investigated dimensions. At the same time, what has been identified in such counties is the greatest fall in inequality, while in counties with a higher level of urbanization, no major changes have been observed. In the material dimension, counties with the highest urbanization level do not show greater changes in QOL because of their relatively balanced and high level of development, which stems from well-functioning local labour markets offering diverse and well-paid jobs (Jażdżewska, 2013). Owing to a relatively easier way of making up basic developmental deficits, a decrease in inequalities has been observed in the counties with an average and low urbanization level. Concerning the social dimension, the situation is different. Highly urbanized areas are diversified in this respect, improving the quality of provided services and creating conditions for taking social action. Counties with an average and low urbanization level, filling gaps in access rather than in quality, diminish the scale of internal differences. These regularities disrupt the intense suburbanisation processes which concentrate around cities and main roads, dramatically changing the scope and nature of developmental processes in rural areas (Reckien & Karecha, 2007). The historical determinants of urban settlement development showing spatial differences mainly in the east–west pattern (low density of weak urban centres vs. well-developed network with strong regional centres) are also not without significance for the observed tendencies (Szymańska & Matczak, 2002).

The third important aspect of spatial differences in QOL in Poland is the functional structure of the counties. The compatibility of QOL with socio-economic growth is also visible (Easterlin, 1974, 2001; Joshua, 2017; Kenny, 1999). Service and industrial-service counties have the highest QOL (and socio-economic development level) and agricultural ones have the lowest (Kurek et al., 2020). The analysis made it possible, in each of the studied dimensions, to rank the counties in terms of QOL from highest to lowest: service > industrial-service > industrial > agro-mixed > agricultural. In the investigated period, the greatest inequalities in the analysed dimensions could be found in industrial-service and agricultural counties, which are generally characterized by large differences in socio-economic development level. A clear increase in inequality in overall QOL was noted in service areas with relatively high internal homogeneity (Mazur & Czapiewski, 2016). Taking material QOL into account, an increase in inequality has been observed in service counties with a relatively high development level and the most diversified labour markets, and definite decreases have appeared in agro-mixed and mono-functional industrial counties. When considering social QOL, there are generally few identified inequalities, and the observed trend is diminishing, which corresponds to the situation regarding the spatial differences of qualitative development factors in the functional pattern of settlement units (Śleszyński & Komornicki, 2016).

## Conclusion

The dynamic economic growth observed in Poland between 2004 and 2018 has not led to substantial changes in spatial differences in QOL at the local level. Persistent differences in QOL are, to a great extent, associated with historically embedded spatial differences in urbanization level and a well-established traditional economic structure of particular territorial units. This example partly confirms the significance of the persistence of institutions in shaping economic and social development, including the quality of life and the specific areas. This is particularly distinct in the region of Wielkopolska (the former Prussian Partition) and Poland’s eastern outskirts (the former Russian Partition). Areas enjoying a relatively high level of economic growth, good infrastructure and inhabited by populations which, in the course of acculturation, took over from Germans positive traits like entrepreneurship, thriftiness, industriousness and lab-abiding. (Wielkopolska), enjoy a high QOL (and considerable QOL growth dynamics). On the other hand, areas with low levels of economic growth and underinvested infrastructure have poor QOL levels and dynamics. The heterogeneity of territorial capital which makes it possible to carry out effective public policies, including programming and the implementation of the Cohesion policy intervention, could play an important role in the further development and improvement of QOL.

The scale of spatial differences in QOL and their deep, historically entrenched embeddedness are the basis for the recommendation to strengthen a place-based policy approach, which requires the decentralization of competences and finances to enhance territorial self-government (Churski et al., 2021; European Commission, 2020). Only with this approach could the material and social dimensions of QOL be effectively consolidated, reducing local spatial contrasts. It is especially important from the point of view of the changing social situation in the last decade in which, as A. Rodríguez-Pose (2017, p. 189) claims:“…persistent poverty, economic decay and lack of opportunities are at the root of considerable discontent in declining and lagging-behind areas the world over…” This is testament to the inefficiency of earlier developmental intervention (Fratesi & Rodríguez-Pose, 2016) and may trigger deeply rooted populism, evident in electoral behaviour in economically less well-off areas, whose development is often “locked” in their history. This makes it even more difficult for these territories to break the closed circle of poverty, which regrettably becomes their permanent feature in many locations worldwide (Bachmann, Sidaway, 2016; Gros, 2016; Rodrik, 2017). The regularities arising from the lack of social approval for the growing development gaps take the form of a certain revenge of the “places that don’t have a future”, which do not want to remain “places that don’t matter” (Rodríguez-Pose, 2017). Counteracting these negative trends requires reinforcement of territory-oriented intervention whose scope will be tailored to effective satisfaction of spatially diverse needs of the inhabitants (Churski et al., 2021). Unfortunatelyongoing preparations for the next financial perspective of the EU 2021–2027 Cohesion policy show that a reduction in the level of programming of European funds at regional and local levels is expected. These propositions reverse the existing decentralist direction of reforms, because a mere 29% of the Cohesion policy means was allocated to Poland (EUR 72.2 bln) for regional operational programmes; this means going back to the situation from 2007. The centralist tendencies weakening self-government in Poland, especially during the COVID-19 pandemic, the economic and social effects of which have emphasized the differences of territorial capital at the local level, may limit the further reduction of differences in terms of QOL or even lead to their increase in terms of the spatial approach.

However, differences in QOL at the local level in today’s Poland are decreasing slightly and gradually. A higher level of urbanization as well as the service and industrial-service functional character of local units are conducive to higher QOL. This trend has been observed in most developing countries (Berdegué et al., 2015; Magel, 2017) and results mainly from a higher level of material conditions, including, first of all, higher incomes and better access to various types of services. These determinants often translate into a greater sense of well-being and a generally higher level of life contentment (Czapińki, 2015). Hence, greater life satisfaction can be found among inhabitants of areas without structural problems (also without high unemployment), and thus often in those with a relatively high socio-economic development level.

## Footnotes

1 In the article, an attempt has been made to analyse the QOL in 380 local units as of 2004 and 2018 (see: part 3 of the article). There are no comparable or credible data for a detailed spatial arrangement like this and two distant moments in time that would allow to describe well-being. Consequently, it is practically impossible to include in the analysis a broad outlook on the QOL, specifically the dimension of wellbeing.

2 Because the principal components are standardized values, each component was moved by vector ${v}_{i}^{^{\prime}}={v}_{i}-\underset{i}{\mathrm{min}}\left\{{v}_{i}\right\}+1/5{s}_{vi}$, thus obtaining positive values, which made it possible to determine the value of the Gini index.

## Declarations

### Data availability

All data with all results are available from the authors.
